# Vice

**Published:** 1914-05

**Authors:** 


### Vice.

As evidence of the growing concern in the vice-problem of the present day is the large number of commissions and societies organized to study the question. Of singular significance is the report of the Pittsburg Moral Efficiency Commission, a municipal investigation by that city, because of its thoroughness and practical conclusions.

"Not until our time," says the commission, "has vice been turned into an organized business, promoted systematically with criminal audacity, having its press agents and lobbyists like other combinations of capital, and resisting fiercely any encroachment upon its privilege of debauching youth for profit. Vice as a business must be wiped out or it will wipe out the civilization that tolerates it."

The commission believes that gain is the chief reason for the existence of panders and procurers and that the Federal White

Slave Act can alone wipe out commercialized vice. It cites as the causes of vice: (1) Economic pressure. (2) Lust of profit. (3) False educational ideals. (4) The spread of luxury. (5) Spiritual apathy. As evidences of its increase it cites: (1) The growth of the social evil. (2) The increase of divorces. (3) Race suicide.

The effect of vice is: (1) Increase in the hidden diseases. (2) Insanity, graft and crime. (3) Artificial social standards. (4) The increase in the cost of living. (5) Penalizing the establishment of the home. (6) Deferring the age of marriage. (7) Increasing promiscuity by the greater intermingling of the sexes in business and education.

The commission further stigmatizes segregation as a "futile compromise," and regulation as in direct opposition to the moral ideas of an American community.

In its recommendation the commission plainly states that virtue can not be enforced by legal enactment, yet uniform laws should be passed by every State to make the giving or receiving of money or other valuable consideration for participation in any act of immorality a felony punishable by imprisonment and fine.

The commission further recommends:

(1) The creation of a non-partisan board of public morals to deal with the social evil.

(2) Legislation providing a minimum wage for women and minors, together with obligatory moral and hygienic protective measures to be used by extensive employers of female labor.

(3) Medical certificates as prerequisites to marriage license.

(4) Rigid medical supervision over inmates of resorts. Reports of all cases of communicable diseases to the department of health.

(5) A municipal hospital with wards for the treatment of certain diseases and the extension of other hospital facilities.

(6) Suppression of harmful advertisements.

(7) Sterilization of criminals and degenerates.

(8) Better censorship of moving picture shows.

(9) More stringent license provision for public dances.

(10) Advocacy of a single moral standard.

